# Electrical Injury-Induced Complete Atrioventricular Block: Is Permanent Pacemaker Required?

**DOI:** 10.1155/2015/158948

**Published:** 2015-12-29

**Authors:** Osman Beton, Tolga Han Efe, Hakki Kaya, Murat Bilgin, Lale Dinc Asarcikli, Mehmet Birhan Yilmaz

**Affiliations:** ^1^Department of Cardiology, Cumhuriyet University, 58140 Sivas, Turkey; ^2^Diskapi Research and Training Hospital, 06110 Ankara, Turkey

## Abstract

A considerable percentage of electrical injuries occur as a result of work activities. Electrical injury can lead to various cardiovascular disorders: acute myocardial necrosis, myocardial ischemia, heart failure, arrhythmias, hemorrhagic pericarditis, acute hypertension with peripheral vasospasm, and anomalous, nonspecific ECG alterations. Ventricular fibrillation is the most common arrhythmia resulting from electrical injury and is the leading cause of death in electrical (especially low voltage alternating current) injury cases. Asystole, premature ventricular contractions, ventricular tachycardia, conduction disorders (various degrees of heart blocks, bundle-brunch blocks), supraventricular tachycardia, and atrial fibrillation are the other arrhythmic complications of electrical injury. Complete atrioventricular block has rarely been reported and permanent pacemaker was required for the treatment in some of these cases. Herein, we present a case of reversible complete atrioventricular block due to low voltage electrical injury in a young electrical technician.

## 1. Introduction

Majority of deaths in adults due to electrical injury are work related, and electrical injury is a frequent cause of work related injury deaths [1]. The primary predictor of injury caused by direct effects of electricity is the extent of current passing through the body [1]. The heart is one of the most susceptible organs to electrical injury which may cause abnormalities such as fatal arrhythmias (asystole, ventricular fibrillation), structural damage, and conduction disturbances [2]. Ventricular fibrillation (VF) is the most common cause of death in electrical (especially low voltage alternating current) injury [3]. Alternating current (AC) is the most frequent cause of electrical injury [1]. Exposure to high voltage current (>1000 volts, AC or direct current) will most likely cause ventricular asystole, whereas exposure to low voltage (<600 volts, mostly AC) will most likely lead to VF [4]. Premature ventricular contractions, ventricular tachycardia, sinus node dysfunction, conduction disorders (varying degrees of heart blocks, bundle brunch blocks), supraventricular tachycardia (mostly sinus tachycardia), and atrial fibrillation have been reported [1, 4, 5]. But complete atrioventricular block (AVB) due to electrical injury has rarely been reported [6–9]. Herein, we present a case of reversible complete atrioventricular block due to low voltage electrical injury in a young electrical technician.

## 2. Case Report

**Figure 1:**
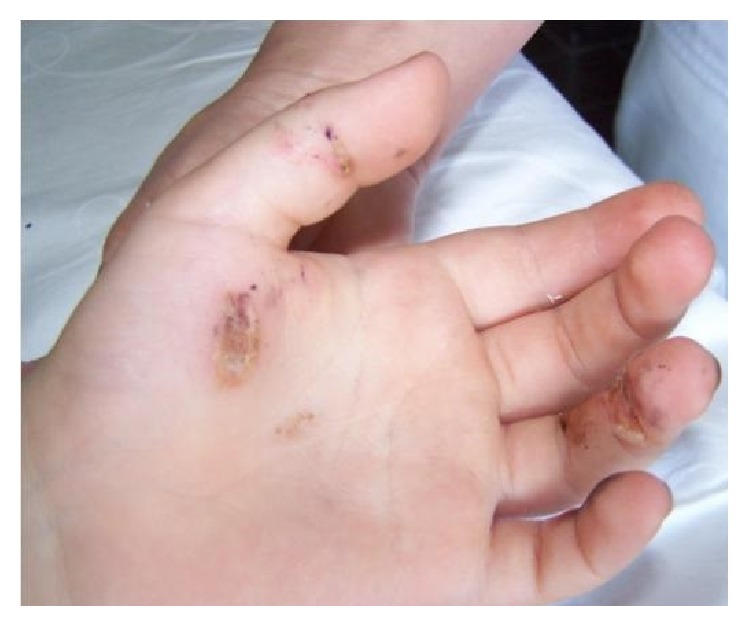
Burn scars of patient as an input site of electrical current in left hand.

**Figure 2:**
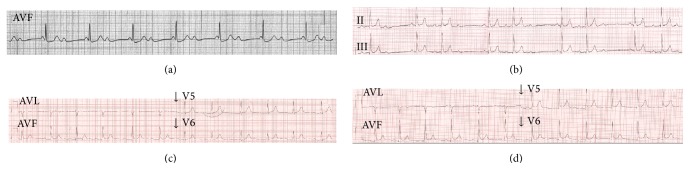
(a) Electrocardiography at admission (complete AV block). (b) Mobitz type I 2nd degree AV block. (c) First degree AV block. (d) Normal sinus rhythm.

A previously healthy 24-year-old male electrical technician was brought to the emergency department after electric injury (380 volts) with shock duration of 10 seconds while he was working on an electric pole. He lost his consciousness transiently (about 20–30 seconds), but he did not fall from the top of the pole. First intervention was performed by ambulance first aid personnel. They found an input site of current in his left hand (Figure 1) and output site in the right gluteal region. Rhythm was complete AVB with a heart rate of 43 at ambulance monitor (Figure 2(a)). Blood pressure was 130/90 mmHg; blood glucose was 135 mg/dL. The patient was brought to emergency department. The rhythm was AV block type I (Figure 2(b)) on the monitor and blood pressure was 125/85 mmHg. But intermittent very short periods of complete AV block were detected on the monitor. He denied smoking, alcohol, or illicit drug use and a history of infection within the past two weeks. Family history was noncontributory. Chest X-ray and echocardiography were completely normal. The results of routine laboratory tests including electrolytes (potassium, calcium, and magnesium) were within normal ranges. Serial blood samples were obtained for detection of myocardial damage. Serum creatine kinase-MB (CK-MB) and troponin I levels were slightly elevated at 40 U/L (normal range: 0–25 U/L) and 0.8 ng/mL (normal: ≤0.06 ng/mL), respectively. The patient was hospitalized in CCU. Temporary pacemaker was not required during follow-up. At third day, ECG changed completely to Mobitz type I AV block and no intermittent period of complete AV block was seen. CK-MB and troponin I levels returned to normal level on the fourth day. The rhythm returned to first degree AV block on day 7 (Figure 2(c)) and normal sinus rhythm on the eighth day (Figure 2(d)). Although there were no chest pain and ischemic ST changes on treadmill exercise test (at 180 bpm) which was performed on the ninth day, coronary angiography (CAG) and electrophysiology study (EPS) were performed before discharge. Normal coronary arteries and normal sinoatrial node and atrioventricular node functions were detected. He was discharged from hospital uneventfully. Holter monitoring records were normal at 3rd month after discharge.

## 3. Discussion

Electrical injury may cause various types of damage to the heart structures such as myocardium, valves, coronary arteries, and conducting tissue [2]. Left ventricular dysfunction, myocardial necrosis, myocardial ischemia, heart failure, hemorrhagic pericarditis, valvular/myocardial rupture, acute hypertension with peripheral vasospasm, and arrhythmias may occur as a consequence of electrical injury [2, 3]. Several different pathophysiologic mechanisms, including direct thermal injury, catecholamine-mediated injuries, coronary artery spasm, ischemia secondary to arrhythmia-induced hypotension, and coronary artery ischemia as part of a generalized vascular injury, have been hypothesized to explain the myocardial and conducting tissue damage due to electrical injury [2, 5].

Postmortem studies observed disseminated focal thermally damaged regions besides hemorrhagic areas in the myocardium of both atria and ventricles at macroscopic examination [3]. Microscopically, various findings, hemorrhagic foci, striated hemorrhages, contraction band necrosis, myocytolysis with coagulative modifications, loss of striations, nuclear disappearance, eosinophilia of the myocytes, and inflammatory reactions, were recorded in the myocardium which was similar to acute myocardial infraction [3]. But coronary arteries and the conduction system were grossly normal in these studies [3]. On the other hand, myofiber break-up changes in myocardial cells were found in ninety percent of cases and hypothesized that these changes could provide the structural substrate necessary to initiate chaotic, electrical asynchronous activity, possibly induced by the passage of abnormal electrical currents [3]. In summary, morphological findings support both direct thermal injury and various degrees of ischemia as the main mechanisms of electrical injury.

Sinus bradycardia and high degree AVB have rarely been reported following electrical injury [1, 4–10]. Sharma et al. reported a young patient with varying degrees of AVB and VF episode after low voltage electrical injury, and spontaneous return to sinus rhythm was observed during follow-up [7]. This case had reversible AVB similar to our case. On the other hand, Hyun et al. reported a young patient with high degree AV block after electrical injury which required permanent pacemaker implantation [8]. Also, a case with electrical injury induced symptomatic bradycardia required permanent pacemaker implantation was reported [9]. In addition, Iino et al. reported transient four different ECG abnormalities: second degree AVB (Wenckebach type), atrial fibrillation, ST-segment depression, and sinus bradycardia in each of four patients after high voltage electrical injury [10]. The reasons for this special vulnerability of the sinus and atrioventricular nodes are not clear [1, 8]. The possible reasons are the following. First, most tissues that develop for the generation and regulation of electrical activity have ionic channels, and control of these channels may be disrupted by exposure to alternating current injury [8]. Second, ischemia or necrosis following electrical injury appears to predominantly affect the distribution of the right coronary artery (supplying both nodes) because of its close proximity to the surface of the chest [1, 8]. Some of these conduction abnormalities may persist and require permanent pacemaker, but some are not. The underlying mechanism is not known so it is hard to predict permanent pacemaker requirement. So, monitoring of these patients seems mandatory. However, symptomatic bradycardias need medications (such as atropine and isoproterenol) or temporary pacemaker may predict permanent pacemaker requirement.

## 4. Conclusion

Electrical injuries can cause various arrhythmias, mostly at the time of the incident. Patients should be followed up in case of ECG abnormality at the initial evaluation and/or loss of consciousness at the onset of injury. Complete AVB is a rare arrhythmic complication of the electrical injury. Besides its rarity, its transient nature should be remembered.
